# Conservation of 5-HT_1A_ receptor-mediated autoinhibition of serotonin (5-HT) neurons in mice with altered 5-HT homeostasis

**DOI:** 10.3389/fphar.2013.00097

**Published:** 2013-08-02

**Authors:** Naozumi Araragi, Boris Mlinar, Gilda Baccini, Lise Gutknecht, Klaus-Peter Lesch, Renato Corradetti

**Affiliations:** ^1^Division of Molecular Psychiatry, Laboratory of Translational Neuroscience, Department of Psychiatry, Psychosomatics, and Psychotherapy, University of WuerzburgWuerzburg, Germany; ^2^Department of NEUROFARBA (Department of Neuroscience, Psychology, Drug Research and Child Health), University of FlorenceFlorence, Italy; ^3^Department of Neurobiology, Institute of Functional Genomics, National Center for Scientific Research (UMR 5203), INSERM U661, University of Montpellier I and IIMontpellier, France

**Keywords:** serotonin transporter, tryptophan hydroxylase-2, knockout, dorsal raphe nucleus, autoinhibition, 5-HT_1A_ receptor

## Abstract

Firing activity of serotonin (5-HT) neurons in the dorsal raphe nucleus (DRN) is controlled by inhibitory somatodendritic 5-HT_1A_ autoreceptors. This autoinhibitory mechanism is implicated in the etiology of disorders of emotion regulation, such as anxiety disorders and depression, as well as in the mechanism of antidepressant action. Here, we investigated how persistent alterations in brain 5-HT availability affect autoinhibition in two genetically modified mouse models lacking critical mediators of serotonergic transmission: 5-HT transporter knockout (*Sert*-/-) and tryptophan hydroxylase-2 knockout (*Tph2*-/-) mice. The degree of autoinhibition was assessed by loose-seal cell-attached recording in DRN slices. First, application of the 5-HT_1A_-selective agonist R(+)-8-hydroxy-2-(di-n-propylamino)tetralin showed mild sensitization and marked desensitization of 5-HT_1A_ receptors in *Tph2*-/- mice and *Sert*-/- mice, respectively. While 5-HT neurons from *Tph2*-/- mice did not display autoinhibition in response to L-tryptophan, autoinhibition of these neurons was unaltered in *Sert*-/- mice despite marked desensitization of their 5-HT_1A_ autoreceptors. When the Tph2-dependent 5-HT synthesis step was bypassed by application of 5-hydroxy-L-tryptophan (5-HTP), neurons from both *Tph2*-/- and *Sert*-/- mice decreased their firing rates at significantly lower concentrations of 5-HTP compared to wildtype controls. Our findings demonstrate that, as opposed to the prevalent view, sensitivity of somatodendritic 5-HT_1A_ receptors does not predict the magnitude of 5-HT neuron autoinhibition. Changes in 5-HT_1A_ receptor sensitivity may rather be seen as an adaptive mechanism to keep autoinhibition functioning in response to extremely altered levels of extracellular 5-HT resulting from targeted inactivation of mediators of serotonergic signaling.

## INTRODUCTION

The brain serotonin (5-HT) system has been implicated in emotion regulation and related psychopathological states, including anxiety, depression, impulsivity, and aggression (reviewed in Lesch et al., 2012). The 5-HT system originates from specified neurons located in distinct nuclei of the brainstem raphe complex. Among them, the dorsal raphe nucleus (DRN) contains the majority of 5-HT neurons and sends projections to various targets in the forebrain. 5-HT neurons in the DRN are known to exhibit spontaneous regular firing activities (Trulson and Jacobs, 1979; Vandermaelen and Aghajanian, 1983). The firing rate of 5-HT neurons is a determinant of 5-HT concentration and thus function in terminal regions, together with local mechanisms (Jacobs and Azmitia, 1992). In waking states, firing of 5-HT neurons is facilitated by noradrenergic input (Levine and Jacobs, 1992). Activity of 5-HT neurons is, in turn, limited by homeostatic negative feedback control exerted by extracellular 5-HT via somatodendritic inhibitory 5-HT_1A_ autoreceptors (Audero et al., 2008 and references therein). The role of 5-HT_1A_ receptors in suppression/regulation of 5-HT neuron firing activity is considered to be relevant to the pathophysiology of disorders of emotion regulation (Pineyro and Blier, 1999; Sharp et al., 2007). The importance of 5-HT_1A_ receptor function is further supported by the presumed mechanism of selective 5-HT reuptake inhibitor (SSRI) antidepressant action (Artigas et al., 1996; Pineyro and Blier, 1999). After acute administration of SSRI, extracellular 5-HT concentrations transiently increase and activate 5-HT_1A_ autoreceptors, inhibiting firing of 5-HT neurons. One criterion of antidepressants’ therapeutic effects is desensitization of these 5-HT_1A_ receptors, leading to a net increase of 5-HT levels. In this context, dysfunction of autoinhibitory 5-HT_1A_ receptors has been proposed as a potential factor contributing to the pathogenesis of emotional disorders. However, studies on 5-HT_1A_ receptor expression in the raphe nuclei of patients with depression measured *in vivo* using positron emission tomography (PET) or in post-mortem brains have yielded contradictory findings: some investigators reported decreased expression (Drevets et al., 1999; Sargent et al., 2000; Arango et al., 2001; Meltzer et al., 2004), while others found enhanced expression (Stockmeier et al., 1998) or no difference compared to controls (Parsey et al., 2006). Moreover, PET imaging data revealed reduced 5-HT_1A_ binding in several brain regions including the raphe complex in panic disorder patients either with or without comorbid depression (Neumeister et al., 2004). To date, most studies concentrated on associations between expression levels of 5-HT_1A_ receptors with depressive disorders and there has been no direct evidence demonstrating how altered 5-HT_1A_ receptor availability translates into the extent of 5-HT neuron autoinhibition. The discrepancies among reports describing a relationship between 5-HT_1A_ receptor expression and depression indicate a need for better understanding of the precise mechanisms linking autoinhibition to 5-HT_1A_ receptor function.

Among various mediators of the brain 5-HT signaling, the 5-HT transporter (SERT, 5-HTT, SLC6A4) plays a central role because (i) it mediates the re-uptake of 5-HT from the extracellular space/synapse and thus terminates the 5-HT signaling and (ii) it is the target of numerous antidepressant drugs which inhibit its action. Carriers of the short variant (s-allele) of the transcriptional control region of the gene encoding SERT (5-HTT gene-linked polymorphic region, 5-HTTLPR), which leads to lower expression and thus a lower amount of SERT protein, are known to convey increased risk for emotional disorders in interaction with environmental factors (reviewed in Canli and Lesch, 2007). On the other hand, tryptophan hydroxylase (TPH) is the rate-limiting enzyme of 5-HT synthesis by converting the essential amino acid L-tryptophan (Trp) into 5-hydroxy-L-tryptophan (5-HTP). 5-HTP is then transformed into 5-HT by aromatic L-amino acid decarboxylase (AADC; Carlsson et al., 1972). While the first isoform TPH1 produces 5-HT in peripheral tissues and the pineal gland, the recently discovered TPH2 isoform is responsible for 5-HT synthesis in the brain (Gutknecht et al., 2009). Variation of the gene coding for TPH2 has been associated with personality traits related to emotional regulation (Gutknecht et al., 2007). Moreover, several polymorphisms in *TPH2*, which had previously been linked to mood disorders, were shown to lead to reduced expression of TPH2 (reviewed in Jacobsen et al., 2012a). Contribution of 5-HT to the regulation of emotion has been further verified by studies on mice with targeted inactivation of either *Sert* or *Tph2*. Indeed, *Sert* knockout (-/-) mice have been shown to display anxiety- and depression-like behaviors (reviewed in Murphy and Lesch, 2008). *Tph2*-/- mice have also been reported to have altered behaviors such as increased conditioned fear responses, aggression, depression-like behaviors, and impairment of maternal care (Savelieva et al., 2008; Alenina et al., 2009; Mosienko et al., 2012; for review, see Lesch et al., 2012).

Here, we investigated firing activity of DRN 5-HT neurons in brain slices obtained from *Sert*-/- mice and *Tph2*-/- mice using loose-seal cell-attached recording configuration. Compared to wildtype (*wt*) controls, *Sert*-/- mice were shown to have ~6- to 10-fold elevated extracellular 5-HT concentrations at baseline in several brain regions including the striatum and the frontal cortex, while heterozygous *Sert*+/- mice were shown to have milder increase, e.g., ~3-fold in the striatum (Fabre et al., 2000; Mathews et al., 2004; Shen et al., 2004). In contrast, *Tph2*-/- mice were reported to display an almost complete depletion of brain 5-HT, while *Tph2*+/- mice showed lower reduction in brain 5-HT, reaching 20–25% in the rostral raphe (Gutknecht et al., 2012). Both knockout mice therefore provide useful models to investigate potential modulation of autoinhibition of 5-HT neuron firing as a function of varying degrees of 5-HT availability in the cellular environment. Moreover, since both mouse lines have extensively been investigated as models for emotional disorders, investigating 5-HT neuron autoinhibitory functions in these mice will facilitate detection of potential alterations in autoinhibition related to disorders of emotion regulation.

In order to mimic *in vivo* 5-HT synthesis in *in vitro* experimental conditions, we applied 5-HT precursors through superfusion of brain slices under recording. Prior to this, we assessed the function of autoinhibitory 5-HT_1A_ receptors by applying their direct agonist. Feasibility of assessing autoinhibition in *in vitro* conditions had been established in previous studies (Liu et al., 2005; Mlinar et al., 2005; Evans et al., 2008; Gutknecht et al., 2012).

## MATERIALS AND METHODS

### ANIMALS

Animal handling followed the European Community guidelines for animal care (DL 116/92, application of the European Communities Council Directive 86/609/EEC) and approved by the local committees. The generation and genotyping procedure of *Tph2*-/- and *Sert*-/- animals were described previously (Bengel et al., 1998; Gutknecht et al., 2008). Animals were housed under a 12 h light/dark cycle (lights on: 08:00–20:00) at ambient temperature of 22 ± 1°C and a relative humidity of 40–50%. Data from *Tph2 wt* and *Sert wt* mice were treated together, since both mouse lines were backcrossed more than 10 generations into a C57BL/6J background and thus considered to have the same genetic background. Data from male and female mice were pooled.

### DRUGS

SR-95531 (gabazine; GABA_A_ receptor antagonist), D-AP5 (NMDA glutamate receptor antagonist), DNQX (AMPA/kainate receptor antagonist) were purchased from Ascent Scientific Ltd (Bristol, UK). *N*-[2-[4-(2-methoxyphenyl)-1-piperazinyl]ethyl]-*N*-2-pyridinylcyclohexanecarboxamide maleate (WAY-100635 maleate; selective 5-HT_1A_ receptor antagonist), CGP-55845 hydrochloride (selective GABA_B_ receptor antagonist), and R(+)-8-hydroxy-2-(di-n-propylamino)tetralin (R(+)-8-OH-DPAT) were purchased from Tocris Bioscience (Bristol, UK). Strychnine (glycine receptor antagonist), Trp, 5-HTP, and L-phenylephrine were obtained from Sigma-Aldrich S.r.l. (Milan, Italy).

### ELECTROPHYSIOLOGICAL RECORDING

Methods used follow those reported previously (Gutknecht et al., 2012). Mice (28–80 days old) were anesthetized with isoflurane and decapitated. The brain was immediately removed, dissected in ice-cold gassed (95% O_2_,5% CO_2_) artificial cerebrospinal fluid (ACSF) containing (in mM): 124 NaCl, 2.75 KCl, 1.25 NaH_2_PO_4_, 1.3 MgCl_2_, 2 CaCl_2_, 26 NaHCO_3_, 11 D-glucose (pH 7.4), and the brainstem was sliced coronally into 200 μm thick slices with a vibratome (DSK-1000; Dosaka Co. Ltd, Kyoto, Japan) and transferred to a multi-well incubation chamber filled with bubbled ACSF at room temperature. After at least 90 min of recovery, the slices were individually transferred into the recording chamber and superfused continuously with gassed, warmed ACSF (34–35°C) at a rate of 2 ml min^-1^. Superfusing ACSF was supplemented with 10 μM phenylephrine to facilitate firing (Vandermaelen and Aghajanian, 1983) and with a mixture of neurotransmitter blockers for glutamate, glycine, and GABA receptors (in μM: 10 DNQX; 20 D-AP5; 10 strychnine; 1 CGP-55845; 10 SR-95531) to functionally isolate the recorded neuron from synaptic input. Neurons were visualized by infrared differential interference contrast video microscopy with a Newicon C2400-07 camera (Hamamatsu, Hamamatsu City, Japan) mounted to an Axioskop microscope (Zeiss, Göttingen, Germany). Recordings were made using an EPC-10 amplifier (HEKA Elektronik, Lambrecht, Germany). Patch pipettes were prepared from thick-walled borosilicate glass on a P-97 Brown-Flaming electrode puller (Sutter Instruments, Novato, CA, USA) and had resistance of 3–6 MΩmega when filled with solution containing (in mM): 125 NaCl, 10 HEPES, 2.75 KCl, 2 CaCl_2_, 1.3 MgCl_2_ (pH 7.4 with NaOH). Loose-seal cell-attached recordings (5–20 MΩmega seal resistance) were acquired continuously in the voltage-clamp mode. Signals were filtered at 3 kHz and digitized at 10 kHz. Pipette potential was maintained at 0 mV. Recordings were aborted if firing rate was sensitive to changes in pipette holding potential or if shapes of action current changed. Data were analyzed using Clampfit 9.2 (Molecular Devices, Sunnyvale, CA, USA).

Neurons with likely serotonergic specification were first targeted according to morphological criteria (Brown et al., 2008): 5-HT neurons are clustered along the midline of the DRN and they have a larger soma (~20–25 μm long-axis diameter) than non-serotonergic neurons (~10–15 μm). Once loose-seal cell-attached recording configuration was established, 5-HT neurons were identified according to electrophysiological criteria (Vandermaelen and Aghajanian, 1983; Allers and Sharp, 2003). Neurons were considered serotonergic if, during at least 5 min-long baseline period at the beginning of the recording displayed slow and steady firing rate (<5 Hz); asymmetric action current with long upstroke to downstroke interval (proportional to action potential half-height width, >0.85 ms). According to these criteria, 250 out of 277 recorded neurons were identified as being serotonergic. Pharmacological experiments were done on 176 presumed serotonergic neurons, whose identity was pharmacologically confirmed based on 5-HT_1A_ receptor-mediated suppression of their firing rate. For all groups of neurons used in pharmacological experiments (**Figures 2–4**), the basal firing rate was matched and proved to be not different after *post hoc* statistical analysis (Kruskal–Wallis test, *p *> 0.7). Since experiments to assess autoinhibition depend on endogenous 5-HT, recordings were made from neurons located at least 50 μm below the slice surface (Mlinar et al., 2005). A single experiment was done in each slice.

For creating concentration–response curves for R(+)-8-OH-DPAT and 5-HTP application, drugs were applied for 10 min and mean firing rates were calculated from the last 1-min segment of each experimental epoch [e.g., baseline, R(+)-8-OH-DPAT 0.1 nM, 0.3 nM, etc.]. Trp was applied for 15 min and mean firing rates were obtained from the last 3-min segment of baseline and Trp application.

### STATISTICAL ANALYSIS

All the statistical tests were performed by GraphPad Prism version 5.04 (GraphPad Software, San Diego, CA, USA). First, normality of data distribution was tested by D’Agostino–Pearson omnibus normality test. When the data were normally distributed, genotype effects were tested by one-way ANOVA [expressed as *F*_(df1, df2)_ values] followed by Tukey’s *post hoc* test. If not, data were analyzed by Kruskal–Wallis test [expressed as *H*_(df)_ values] with Dunn’s *post hoc* test. For testing effects of Trp in comparison to respective baseline, data (% change in firing rates) were analyzed by Wilcoxon signed rank test (two-tailed). In all cases, *p *< 0.05 was considered statistically significant.

## RESULTS

### COMPARISON OF BASAL FIRING RATES ACROSS GENOTYPES

In the absence of precursor supplementation (Trp or 5-HTP), and in the presence of receptor blockers for glutamate, GABA, and glycine receptors, the basal firing of 5-HT neurons in slices is relieved from the autoinhibitory control of endogenous 5-HT (Mlinar et al., 2005) and local action of major neurotransmitters. In these conditions of pharmacological isolation, the basal firing activity of 5-HT neurons reflects their *intrinsic* pacemaker activity, a characteristic that is difficult to study *in vivo*, where the firing activity is under control of both autoinhibition and synaptic input.

**FIGURE 1:**
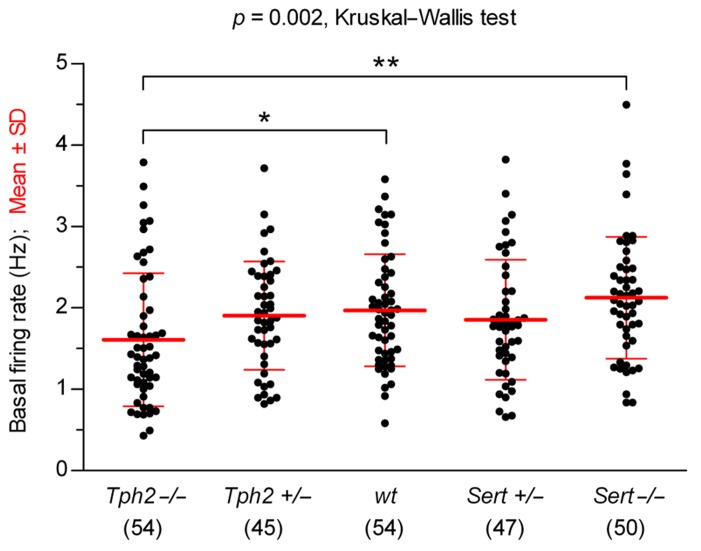
**Comparison of 5-HT neuron basal firing rates across genotypes.** Data are shown as mean ± SD (number of cells shown in parentheses). Analysis of data using Kruskal–Wallis test revealed statistically significant differences among groups [*H*_(4)_ = 16.67, *p* = 0.002]. Dunn’s multiple comparison *post hoc* test resulted in being significant for *Sert*-/- vs. *Tph2*-/- (**p < 0.01) and *wt* vs. *Tph2*-/- (*p < 0.05).

We compared the basal firing rates recorded before 5-HT precursor or 5-HT_1A_ receptor agonist application, across genotypes (**Figure 1**). Overall, 5-HT neurons showed typical regular pacemaker activity and firing rates similar to *wt* controls [in Hz: *Tph2*-/-, 1.61 ± 0.82 (*n* = 54); *Tph2*+/-, 1.90 ± 0.66 (*n* = 45); *wt*, 1.97 ± 0.69 (*n* = 54); *Sert*+/-, 1.85 ± 0.74 (*n* = 47); *Sert*-/-, 2.12 ± 0.75 (*n* = 50); mean ± SD; *n* = number of recorded neurons], except for *Tph2*-/- in which the firing rate was slightly, but significantly slower than in *wt* controls (*p *< 0.05, Kruskal–Wallis test followed by Dunn’s multiple comparison test). These data show that basic electrophysiological properties underlying the typical pacemaker activity of 5-HT neurons are maintained regardless of genetic inactivation of *Tph2* or *Sert*.

### COMPARISON OF 5-HT_1A_ RECEPTOR SENSITIVITY ACROSS GENOTYPES

**FIGURE 2:**
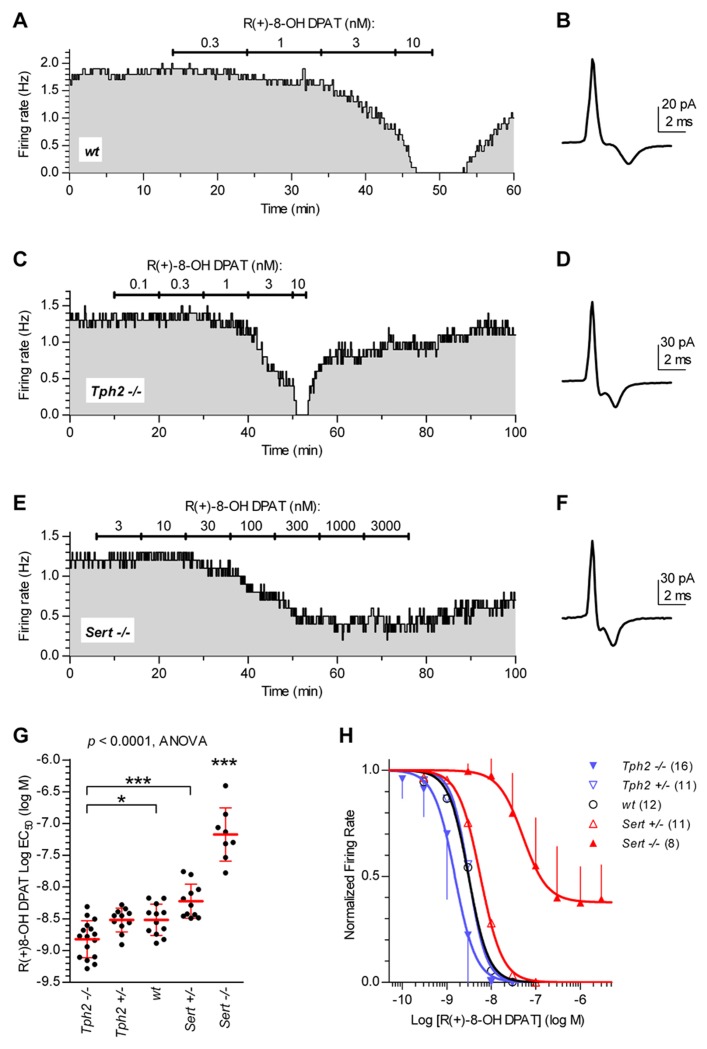
**Sensitivity of 5-HT neurons to R(+)-8-OH-DPAT differs across genotypes.** Time courses of firing rate changes in response to increasing concentrations of R(+)-8-OH-DPAT of individual 5-HT neurons in brain slices obtained from *wt* **(A,B)**, *Tph2*-/- **(C,D)**, and *Sert*-/- mice **(E,F)**. Traces show action current of corresponding neurons recorded. **(G)** Dots represent log EC_50_ of concentration–responses from individual experiments. Red lines report mean ± SD of values. One-way ANOVA followed by Tukey’s multiple comparison test showed statistically significant differences across genotypes [*F*_(4,53)_ = 48.38, *p < *0.0001]. Asterisks indicate level of statistical significance between the indicated genotypes (for *Sert*-/-, vs. all the other four genotypes): ***p < 0.001, *p < 0.05. **(H)** Average concentration–response curves obtained from all the experiments. Each data point corresponds to the mean from several neurons (numbers in parentheses). For the sake of clarity, error bars are shown only for *Sert*-/- mice and *Tph2*-/- mice in a single direction. Data are normalized on average baseline firing rates recorded before R(+)-8-OH-DPAT application. Note that, curves for *Sert*-/- mice did not achieve full inhibition of firing (see **E**).

Since 5-HT neuron autoinhibition is mediated by 5-HT_1A_ receptors, we investigated the functional response of 5-HT neurons to the 5-HT_1A_ receptor agonist R(+)-8-OH-DPAT in different genotypes. **Figure 2** illustrates typical experiments in which increasing concentrations of R(+)-8-OH-DPAT were applied in slices from *wt* controls (**Figures 2A,B**), *Tph2*-/- (**Figures 2C,D**), and *Sert*-/- mice (**Figures 2E,F**). Application of R(+)-8-OH-DPAT reduced the firing rate of 5-HT neurons in a concentration-dependent manner, but with different effectiveness across genotypes, as shown by the comparison of log EC_50_ values obtained for each single neuron tested (log EC_50_ mean ± SD): *Tph2*-/-, -8.82 ± 0.29 (*n* = 16); *Tph2*+/-, -8.52 ± 0.19 (*n* = 11); *wt*, -8.52 ± 0.25 (*n* = 12); *Sert*+/-, -8.22 ± 0.27 (*n* = 11); *Sert*-/-, -7.17 ± 0.42 (*n* = 8; **Figure 2G**). Differences across genotypes were statistically significant [*F*_(4,53)_ = 48.38, *p < *0.0001, one-way ANOVA]. Compared to *wt* controls, the response to application of R(+)-8-OH-DPAT resulted in slightly higher effectiveness of the agonist in *Tph2*-/- mice (*p < *0.05) and very weak effectiveness in *Sert*-/- mice (*p < *0.001). Although a small decrease in the sensitivity of 5-HT neurons was present also in *Sert*+/- mice, no statistically significant differences in log EC_50_ values were found for both *Tph2*+/- and *Sert*+/- vs. *wt *control mice, indicating that limited impairment of 5-HT synthesis and re-uptake did not result in relevant changes of 5-HT_1A_ autoreceptor sensitivity to R(+)-8-OH-DPAT. **Figure 2H** shows concentration–response curves fitted for each group on mean data obtained from the individual experiments shown in **Figure 2G**. It should be noted that in *Sert*-/- neurons, R(+)-8-OH-DPAT did not produce maximal inhibition of firing (see **Figure 2E**). Nevertheless, the average maximal inhibition was 60% compared to the other genotypes and the mean log value of concentrations producing an actual 50% decrease in firing of *Sert*-/- neurons was -6.91 ± 0.08 (*n* = 8), which did not affect the level of significance for decreased sensitivity of 5-HT_1A_ receptors shown in **Figure 2G**. Collectively, these data show that the sensitivity of 5-HT_1A_ receptors to agonist activation is markedly affected in *Sert*-/-.

### ESTIMATION OF AUTOINHIBITION EXERTED BY ENDOGENOUS 5-HT ACROSS GENOTYPES

After assessing responsiveness to direct activation by the 5-HT_1A_ receptor agonist R(+)-8-OH-DPAT in the different genotypes, we investigated how specific genetic alterations translate into inhibition of 5-HT neuron activity by endogenous 5-HT. Once synthesis of 5-HT is restored in slices by supplementation of 5-HT precursors, the extent of autoinhibition in the different genotypes will depend on the balance between the level of extracellular 5-HT determined by the alteration of homeostatic mechanisms introduced by genetic manipulation and 5-HT_1A_ receptor sensitivity characteristic of each genotype.

Thus, we studied autoinhibition exerted by endogenous 5-HT, when *de novo* synthesis was restored in slices by supplementation of Trp or 5-HTP. Trp was used to estimate the extent of autoinhibition in respect to bioavailability of the natural precursor (Mlinar et al., 2005). 5-HTP was used to bypass the constraint in 5-HT synthesis produced by the rate-limiting enzyme Tph2. This allows reaching extracellular 5-HT concentrations greater than with Trp and permits quantification of the overall capacity of 5-HT neuron autoinhibition in different genotypes, including *Tph2*-/- mice.

**FIGURE 3:**
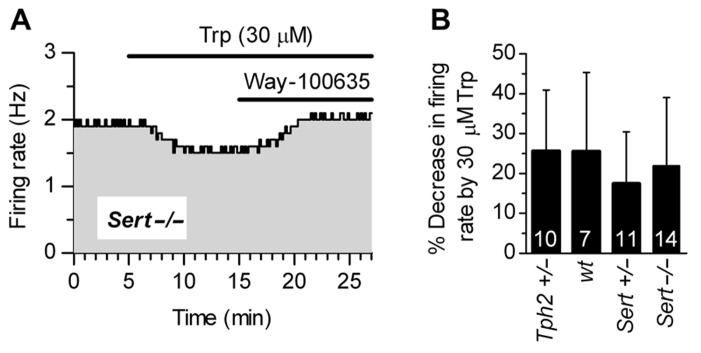
** Autoinhibition by endogenous 5-HT synthesized from Trp is conserved across genotypes.** **(A)** Trp (30 μM) produced a 5-HT_1A_ receptor-mediated decrease in the firing rate of a 5-HT neuron from *Sert*-/- mice. Application of a selective 5-HT_1A_ receptor antagonist, WAY-100635 (50 nM; representative of three experiments) fully antagonized Trp effect, confirming that autoinhibition was mediated by 5-HT_1A_ autoreceptors. **(B)** Application of 30 μMTrp significantly suppressed firing activity of DRN serotonergic neurons in all the genotypes tested (*p < *0.05; Wilcoxon signed rank test). Comparison of the effect of Trp among genotypes revealed no statistically significant differences among genotypes [*H*_(3)_ = 3.336, *p* = 0.3427; Kruskal–Wallis test]. Data are shown as mean ± SD. The number of neurons recorded for each genotype is shown at the bottom of the histograms.

**Figure 3A** shows that supplementation of Trp (30 μM) produced a decrease in firing rates of *Sert*-/- 5-HT neurons, an effect fully antagonized by WAY-100635, a selective 5-HT_1A_ receptor neutral antagonist (Corradetti et al., 1998). This demonstrates that 5-HT_1A_ receptor-mediated autoinhibition is present in *Sert*-/- mice. As shown in **Figure 3B**, 30 μM Trp significantly decreased firing rates of 5-HT neurons to a similar extent in all the genotypes tested (in % ± SD): *Tph2*+/-, 25.62 ± 15.37 (*n* = 10); *wt*, 25.55 ± 19.87 (*n* = 7); *Sert*+/-, 17.51 ± 12.99 (*n* = 11); *Sert*-/-, 22.03 ± 17.00 (*n* = 14). In all cases, the decrease in firing rates was significantly different from zero (*p < *0.05; Wilcoxon signed rank test). Furthermore, responses to application of Trp were not statistically different across four genotypes [H_(3)_ = 3.336, *p* = 0.3427; Kruskal–Wallis test]. These data show that autoinhibition of DRN 5-HT neurons by endogenous 5-HT is conserved in all the genotypes to a similar level, irrespective of the genetic alteration.

To quantify the extent to which each genotype conserved the capacity to (auto)inhibit 5-HT neuron firing in response to different extracellular concentrations of endogenous 5-HT, we investigated the functional response of 5-HT neurons to 5-HTP in different genotypes.

**FIGURE 4:**
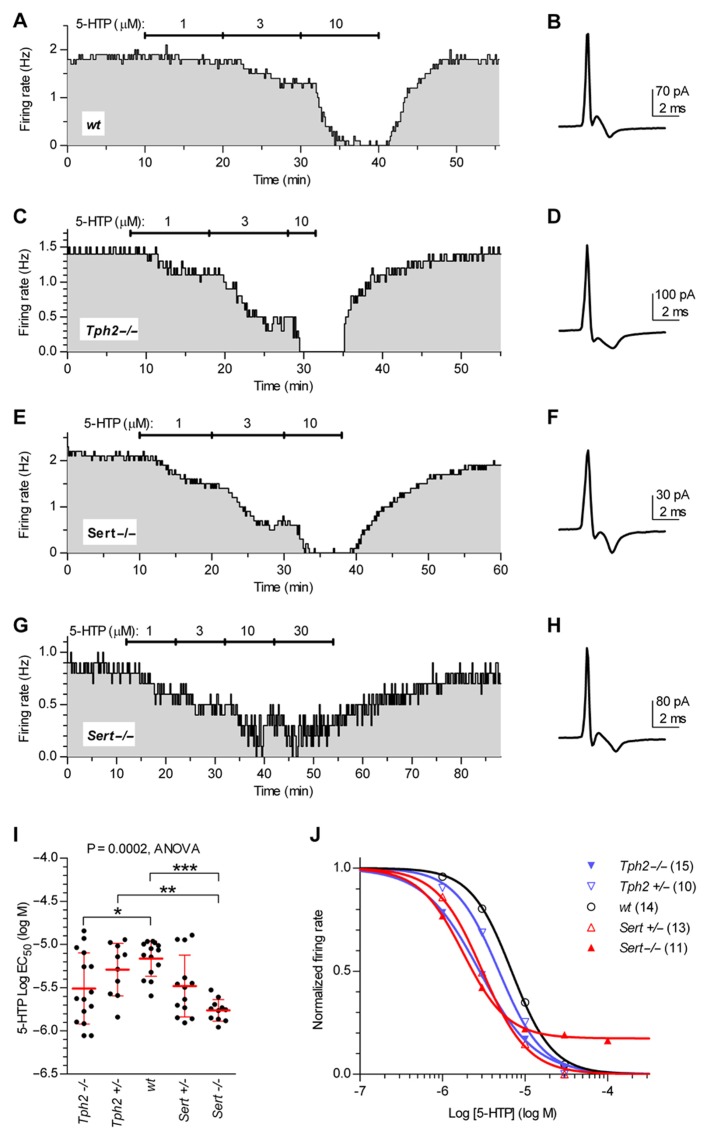
**Quantification of autoinhibition capacity of 5-HT neurons across genotypes by concentration–response curves for 5-HTP.** Time courses of 5-HT neuron firing rate changes in response to increasing concentrations of 5-HTP in brain slices obtained from *wt* controls **(A,B)**, *Tph2*-/- **(C,D)**, and *Sert*-/- mice **(E–H)**. Traces show action current of corresponding neurons recorded. **(I)** Dots represent log EC_50_ of concentration–responses from individual experiments. Red lines report mean ± SD of values. One-way ANOVA followed by Tukey’s multiple comparison test showed statistically significant differences [*F*_(4,58)_ = 6.723, *p* = 0.0002] Asterisks indicate level of statistical significance between the indicated genotypes: ***p < 0.001, **p < 0.01, *p < 0.05. **(J)** Average concentration–response curves obtained from all the experiments. Each data point corresponds to the mean from several neurons (numbers shown in parentheses). For the sake of clarity, error bars are omitted. Data are normalized on average baseline firing rates recorded before 5-HTP application.

**Figure 4** illustrates firing rate changes of 5-HT neurons in response to increasing concentrations of 5-HTP in brain slices obtained from *wt* controls (**Figures 4A,B**), *Tph2*-/- (**Figures 4C,D**), and *Sert*-/- mice (**Figures 4E–H**). Application of 5-HTP reduced the firing rate of 5-HT neurons in a concentration-dependent manner, but with different effectiveness across genotypes [one-way ANOVA, *F*_(4,58)_ = 6.723, *p* = 0.0002], as shown by the comparison of log EC_50_ values obtained for each single neuron tested (log EC_50_ mean ± SD): *Tph2*-/-, -5.51 ± 0.41 (*n* = 15); *Tph2*+/-, -5.29 ± 0.30 (*n* = 10); *wt*, -5.17 ± 0.20 (*n* = 14); *Sert*+/-, -5.48 ± 0.36 (*n* = 13); *Sert*-/-, -5.76 ± 0.12 (*n* = 11; **Figure 4I**). Interestingly, the sensitivity to the effects of endogenous 5-HT synthesized from 5-HTP was increased both in *Tph2*-/- (*p < *0.05) and *Sert*-/- (*p < *0.001) mice compared to *wt* controls. **Figure 4J** shows concentration–response curves fitted for each group on mean data obtained from the individual experiments depicted in **Figure 4I**.

Whereas a stronger autoinhibitory response to 5-HTP in *Tph2*-/- mice is consistent with the observed increase in sensitivity of 5-HT_1A_ receptors to agonist activation, a similar increase in *Sert*-/- mice is unexpected in the presence of decreased sensitivity to R(+)-8-OH-DPAT. We suggest that, due to the absence of 5-HT re-uptake, in *Sert*-/- mice the extracellular 5-HT neosynthesized from 5-HTP attains higher levels than in *wt *control mice, leading to this apparent increase in response. Collectively, these results demonstrate that the changes in sensitivity to direct activation of 5-HT_1A_ receptors cannot directly be translated into the expected changes in autoinhibition exerted by endogenous 5-HT.

## DISCUSSION

In the present study, we have investigated the relationship between the sensitivity of 5-HT_1A_ receptors and the concomitant degree of autoinhibition of 5-HT neurons in a panel of genetically modified mice characterized by impairment of cellular mechanisms crucial for homeostatic control of extracellular 5-HT levels (i.e., 5-HT synthesis and 5-HT re-uptake). *In vivo*, these genetic manipulations are likely to produce lifelong persistent modifications of 5-HT levels ranging from the absence of 5-HT in *Tph2*-/- mice (Savelieva et al., 2008; Alenina et al., 2009; Gutknecht et al., 2012) to a substantial increase in extracellular 5-HT levels in *Sert*-/- mice (Fabre et al., 2000; Mathews et al., 2004; Shen et al., 2004). The consequences of genetic alterations are maintained *in vitro*. This provides a set of conditions in which the relationship between the sensitivity of 5-HT_1A_ receptors and the autoinhibitory response of 5-HT neurons exerted by endogenous 5-HT could be quantitatively compared.

The major finding of the present study is that substantial and persistent alterations in 5-HT homeostasis produced changes in the sensitivity of 5-HT_1A_ receptors that did not translate in measurable changes of autoinhibitory regulation of 5-HT neuron firing. In particular, *Sert*-/- mice showed a marked subsensitivity of 5-HT_1A_ receptors, but displayed a normal capacity of autoinhibition. Interestingly, the sensitivity of 5-HT_1A_ receptors of both *Sert*+/- and *Tph2*+/- mice proved to be similar to that of *wt* control mice, showing that mild change in extracellular 5-HT levels is neither a strong stimulus for 5-HT_1A_ receptor adaptive changes in sensitivity, nor does it detectably affect autoinhibition.

In previous studies under similar recording conditions as used in this work, raphe slices showed substantial depletion of 5-HT in the absence of 5-HT precursors (Liu et al., 2005; Mlinar et al., 2005). *In vitro*, 5-HT content, together with 5-HT_1A_ receptor-mediated autoinhibition, can be restored by supplementation of Trp (Liu et al., 2005; Mlinar et al., 2005; Evans et al., 2008; Gutknecht et al., 2012). This allowed electrophysiological, quantitative, assessment of the modifications in sensitivity of 5-HT_1A_ receptors produced by altered 5-HT homeostasis *in vivo* and estimation of the functional state of autoinhibition when *de novo* synthesis of 5-HT was restored in slices.

### GENETIC MANIPULATIONS DO NOT AFFECT PACEMAKER CHARACTERISTICS OF 5-HT NEURONS

The pacemaker properties of serotonergic neurons measured in slices in the virtual absence of endogenous 5-HT neosynthesis, hence of autoinhibition, were not substantially altered by genetic manipulation itself, as we observed similar baseline firing rates among genotypes, except for *Tph2*-/- mice, which had slightly lower baseline firing rates compared to the other genotypes. This shows that the basic characteristics of intrinsic pacemaker firing activity of 5-HT neurons are preserved independently from genetic manipulations that altered 5-HT homeostatic regulation. The small decrease in baseline firing rates observed in *Tph2*-/- mice may indicate that, in the chronic absence of 5-HT, neurons adapt their membrane properties, e.g., conductance, to compensate for absent autoinhibition and homeostatically keep pacemaker firing activity constant. The mechanism(s) underlying this adaptation is currently under investigation. It should be noted that the basal firing rate recorded under our experimental conditions, i.e., *in vitro*, results from the interplay of ion conductances responsible for pacemaking activity and likely do not correspond to the “basal” firing rate recorded *in vivo* (e.g., Gobbi et al., 2001; Bouali et al., 2003; see below) which is under the control of 5-HT_1A_ receptor-mediated autoinhibition in all genotypes (see **Figure 3**), except in *Tph2*-/- mice (Gutknecht et al., 2012).

### LIFELONG EXPOSURE OF 5-HT NEURONS TO VARYING 5-HT LEVELS RESULTS IN CHANGES IN THE SENSITIVITY OF SOMATODENDRITIC 5-HT_1A_ RECEPTORS

Previous studies showed adaptive decrease in sensitivity of 5-HT_1A_ receptors in *Sert*-/- mice (Lanfumey et al., 2000; Mannoury la Cour et al., 2001; Bouali et al., 2003). Our study extends the investigation to the opposite extreme, i.e., *Tph2*-/- mice, which are devoid of 5-HT and show a small, but significant increase in 5-HT_1A_ receptor sensitivity. This is consistent with neurochemical data showing an increase in 5-HT_1A_ receptor density in the raphe (Gutknecht et al., 2012).

In *Sert*-/- mice, we found a decrease in the maximal response to R(+)-8-OH-DPAT (~40%) and a similar reduction of autoinhibitory capacity as revealed by concentration–response curves with 5-HTP. This may reflect a downregulation of 5-HT_1A_ receptors due to lifelong exposure to increased stimulation by 5-HT or the emergence of a still-unknown adaptive mechanism directed to counteract increased autoinhibition exerted by high levels of extracellular 5-HT *in vivo*. In spite of the decrease, however, the remaining autoinhibition capacity of 5-HT neurons largely exceeded the magnitude of physiological autoinhibition produced by 5-HT when its synthesis was restored by Trp (see below).

Taken together, our data indicate that the level of 5-HT_1A_ receptor sensitivity of 5-HT neurons is inversely correlated with extracellular levels of 5-HT *in vivo*, at least in extreme conditions as represented by *Tph2*-/- and *Sert*-/- mice.

### AUTOINHIBITION OF 5-HT NEURONS BY ENDOGENOUS 5-HT IS CONSERVED IN THE PHYSIOLOGICAL RANGE, REGARDLESS OF THE SENSITIVITY OF 5-HT_1A_ RECEPTORS

When the level of autoinhibition restored by Trp supplementation in slices from all the genotypes (except *Tph2*-/-) was measured, this resulted in being similar, irrespective of the sensitivity of 5-HT_1A_ receptors measured in each genotype. Notably, *Sert*-/- showed greatly decreased sensitivity to the agonist but normal autoinhibition, as estimated by Trp challenge. Accordingly, the autoinhibitory effect of endogenous 5-HT synthesized *de novo* from 5-HTP proved to be not decreased in all the mutants compared with *wt* controls, including *Tph2*-/- in which the absence of Tph2 was bypassed by 5-HTP. It should be noted that in *Sert*-/- mice the maximal inhibitory response was slightly decreased (~20%) in agreement with the reduced maximal response to the agonist, but the substantial residual inhibition capacity is apparently sufficient to produce a physiological level of autoinhibition as shown by Trp experiments. In conclusion, these data indicate that the marked subsensitivity of 5-HT_1A_ receptors observed in *Sert*-/- does not translate in the loss of normal autoinhibition capacity of 5-HT neurons.

Although counterintuitive, this notion is consistent with the observation that, *in vivo*, the firing rate of 5-HT neurons is not increased in *Sert*-/-, but similar to or even lower (Gobbi et al., 2001; Bouali et al., 2003) than that of *wt *controls, thus indicating that *in vivo *subsensitivity of 5-HT_1A_ receptors in *Sert*-/- mice does not relieve 5-HT neurons from autoinhibition. Furthermore, Fox et al. (2010) reported that in these mice antagonism of 5-HT_1A_ receptors by WAY-100635 resulted in the appearance of greater frequency of 5-HT_2A_ receptor-mediated head twitches than in *wt *controls. This suggests that the relief from autoinhibition, hence the increase in 5-HT neuron firing, produces an increase in 5-HT release sufficient to produce this 5-HT_2A_-mediated behavioral effect (Willins and Meltzer, 1997), even in the presence of partial desensitization of 5-HT_2A_ receptors (Rioux et al., 1999; Li et al., 2003; Qu et al., 2005).

### IMPLICATIONS OF THE DIVERGENCE BETWEEN SENSITIVITY TO R(+)-8-OH-DPAT AND 5-HT NEURON AUTOINHIBITION

The crucial role of somatodendritic 5-HT_1A_ receptors in regulating the firing rate of 5-HT neurons, hence the functional state of 5-HT system, has attracted interest in the attempt to infer the degree of activity of these neurons in pathological conditions of humans and in behavioral experiments of rodents. The present work may help to better understand the limits in the interpretation of the functional state of 5-HT system based on measurements of density/sensitivity of 5-HT_1A_ receptors of 5-HT neurons. Furthermore, since the knockout mice used in this investigation may model different risk factors (i.e., *TPH2* and *SERT* polymorphisms) for anxiety disorders and depression, our data showing that autoinhibition is not impaired in these mutants may provide a reference background for the interpretation of behavioral responses in these mice in the context of human psychopathology. For instance, functional autoinhibition in patients with depression were indirectly inferred from 5-HT_1A_ receptor imaging studies in the raphe (Drevets et al., 2007; Savitz et al., 2009). Overall, however, these studies failed to clarify whether the depression-related changes in 5-HT_1A_ receptor binding are genetically or environmentally driven during development, thus causative of the disorder, or whether they are simply an adaptation to acutely increased or decreased serotonergic transmission (Savitz et al., 2009).

Contradicting results were also gathered in the attempt to associate *SERT* polymorphisms with changes in the level of 5-HT_1A_ receptor expression/density. David et al. (2005) reported that carriers of the 5-HTTLPR s-allele had lower 5-HT_1A_ receptor binding potential in all the brain regions investigated compared to individuals homozygous for the l-allele. On the contrary, Lee et al. (2005) found that s-carriers had higher 5-HT_1A_ binding than ll-individuals in pregenual and subgenual cingulate cortex regions while in other regions, including the DRN, no difference was detected. More recently, Borg et al. (2009) could not reveal any differences in 5-HT_1A_ receptor density between carriers and non-carriers of the 5-HTTLPR s-allele and concluded that functional consequences of 5-HTTLPR are not likely to be mediated by differences in 5-HT_1A_ expression. Our results showing that 5-HT system autoinhibition is not reduced in mice with impaired Sert function even in the presence of altered 5-HT_1A_ receptor sensitivity would support this conclusion.

A second implication of our results involves the possibility to infer the degree of 5-HT system autoinhibition from functional assays using activation of 5-HT_1A_ receptors with direct agonists, in patients or in animal models. For example, one of the most consistent findings among depressed patients is their blunted hypothermia in response to 5-HT_1A_ receptor direct agonists (Lesch et al., 1990; Lesch, 1991; Jacobsen et al., 2012b and references therein). Such responses are usually ascribed to desensitization of somatodendritic 5-HT_1A_ receptors (reviewed in Jacobsen et al., 2012a). Our data suggest that, whereas blunted hypothermic response to direct agonists is likely to reflect subsensitivity of 5-HT_1A_ receptors in these patients, this decrease in response cannot directly be correlated to functional consequences that entail reduced autoinhibition and increase in the basal firing rate of 5-HT neurons.

On the other hand, the finding that 5-HT neurons in Tph2- and Sert-deficient mice display normal responsiveness to Trp and/or 5-HTP regarding autoinhibition of 5-HT neuron firing would support the use of Trp (or 5-HTP) as an appropriate challenge to test the functional state of 5-HT system in clinical settings and to reveal the involvement of altered autoinhibition in human psychopathology. Indeed, 5-HTP challenge has been successfully applied to reveal functional consequences dependent on 5-HTTLPR variation in humans (Maron et al., 2004).

Finally, the striking divergence between sensitivity to R(+)-8-OH-DPAT and 5-HT neuron autoinhibition in *Sert*-/- suggests the possibility that sustained increase in 5-HT levels by stressors or pharmacological treatments (e.g., SSRIs) may result in 5-HT_1A_ receptor subsensitivity, not accompanied by functional impairment of 5-HT neuron firing autoregulation. For instance, the rapid decrease in 5-HT_1A_ receptor sensitivity found in DRN 5-HT neurons following chronic ultramild stress and stressful uncontrolled environmental conditions is apparently not correlated with an increase in 5-HT system activity and has been suggested to be an adaptive mechanism to compensate for 5-HT fluctuations produced by stressful events (Laaris et al., 1999; Lanfumey et al., 1999). Interestingly, *in vivo* recording after chronic unpredictable stress in rats showed that the reduced ability of 8-OH-DPAT to inhibit 5-HT neuron firing was accompanied by a decrease in firing rate of DRN 5-HT neurons (Bambico et al., 2009), indicating that functional autoinhibition may be preserved in spite of 5-HT_1A_ receptor desensitization. Furthermore, desensitization of autoinhibitory 5-HT_1A_ receptors occurring with chronic SSRI administration (Le Poul et al., 2000; Hensler, 2002; Castro et al., 2003) has been proposed as a mechanism for 5-HT neurons to escape the sustained autoinhibition produced by the increase in 5-HT in raphe nuclei by blockade of Sert and to represent an important step to achieve enhanced therapeutic effects of SSRIs (Artigas et al., 1996). On the other hand, Richardson-Jones et al. (2010) showed that desensitization of 5-HT_1A_ autoreceptors is not sufficient for antidepressants to convey their efficacy, indicating dissociation between desensitization of 5-HT_1A_ autoreceptors and behavioral effects of chronic SSRI treatment. Thus, desensitization of 5-HT_1A_ autoreceptors appears rather to be an adaptive mechanism to neutralize elevated extracellular 5-HT levels, and not a primary factor leading to behavioral alteration.

Under a functional perspective, however, dynamic changes in the sensitivity/expression of 5-HT_1A_ receptors appear to be crucial to fulfill the requirements for physiological homeostasis of 5-HT system functioning. Thus, any impairment of adaptive mechanisms of 5-HT_1A_ receptors in response to sustained changes in 5-HT levels, or constitutive alteration of their expression even in the absence of altered 5-HT levels *in vivo*, becomes a potential source of pathological consequences. In fact, genetically induced overexpression of somatodendritic 5-HT_1A_ receptors in mice has been shown to produce autonomic dysregulation (Audero et al., 2008), behavioral alterations, and decreased response to antidepressant drugs (Richardson-Jones et al., 2010). In humans, the C(-1019)G 5-HT_1A_ promoter polymorphism leading to 5-HT_1A_ receptor overexpression is proposed to represent a risk factor for depression (Lemonde et al., 2003; Strobel et al., 2003; Rothe et al., 2004; reviewed in Albert and Francois, 2010) and response to antidepressant drugs (reviewed in Albert, 2012).

In conclusion, our data reveal that 5-HT neuron autoinhibition is similar in all *Tph2* and *Sert *genotypes studied, regardless of the different sensitivity of their somatodendritic 5-HT_1A_ receptors to R(+)-8-OH-DPAT. This suggests that adaptive changes in receptor sensitivity occur to compensate for variable extracellular 5-HT levels in different genotypes to homeostatically conserve autoinhibition in a physiological range. Thus, it appears that response to 5-HT_1A_ agonists *per se* is not always sufficient for evaluating the functional state of the 5-HT system, for which Trp and/or 5-HTP challenges may provide more informative data, both in clinical and animal experimental settings.

## Conflict of Interest Statement

The authors declare that the research was conducted in the absence of any commercial or financial relationships that could be construed as a potential conflict of interest.

## Abbreviations

DRN: dorsal raphe nucleus; 5-HT, serotonin; 5-HTP, 5-hydroxy-L-tryptophan; R(+)-8-OH-DPAT, R(+)-8-hydroxy-2-(di-n-propylamino)tetralin; Sert, serotonin transporter; Tph2, tryptophan hydroxylase-2; Trp, L-tryptophan.
